# The relative performance of geometric morphometrics and linear‐based methods in the taxonomic resolution of a mammalian species complex

**DOI:** 10.1002/ece3.9698

**Published:** 2023-03-28

**Authors:** Pietro Viacava, Simone P. Blomberg, Vera Weisbecker

**Affiliations:** ^1^ School of Biological Sciences The University of Queensland St Lucia QLD Australia; ^2^ College of Science and Engineering Flinders University Adelaide SA Australia; ^3^ Centre of Excellence for Australian Biodiversity and Heritage Australian Research Council Canberra ACT Australia; ^4^ Australian National Wildlife Collection CSIRO National Research Collections Australia Canberra ACT Australia

**Keywords:** allometry, cryptic species, geometric morphometrics, linear discriminant analysis, linear morphometrics, shape variation, taxonomy

## Abstract

Morphology‐based taxonomic research frequently applies linear morphometrics (LMM) in skulls to quantify species distinctions. The choice of which measurements to collect generally relies on the expertise of the investigators or a set of standard measurements, but this practice may ignore less obvious or common discriminatory characteristics. In addition, taxonomic analyses often ignore the potential for subgroups of an otherwise cohesive population to differ in shape purely due to size differences (or allometry). Geometric morphometrics (GMM) is more complicated as an acquisition technique but can offer a more holistic characterization of shape and provides a rigorous toolkit for accounting for allometry. In this study, we used linear discriminant analysis (LDA) to assess the discriminatory performance of four published LMM protocols and a 3D GMM dataset for three clades of antechinus known to differ subtly in shape. We assessed discrimination of raw data (which are frequently used by taxonomists); data with isometry (i.e., overall size) removed; and data after allometric correction (i.e., with nonuniform effects of size removed). When we visualized the principal component analysis (PCA) plots, we found that group discrimination among raw data was high for LMM. However, LMM datasets may inflate PC variance accounted in the first two PCs, relative to GMM. GMM discriminated groups better after isometry and allometry were removed in both PCA and LDA. Although LMM can be a powerful tool to discriminate taxonomic groups, we show that there is substantial risk that this discrimination comes from variation in size, rather than shape. This suggests that taxonomic measurement protocols might benefit from GMM‐based pilot studies, because this offers the option of differentiating allometric and nonallometric shape differences between species, which can then inform on the development of the easier‐to‐apply LMM protocols.

## Graphical abstract

Morphology‐based taxonomic research frequently applies LMM in skulls to quantify species distinctions but the choice of which measurements to collect generally relies on the expertise of the investigators, a practice that may ignore less obvious discriminatory characters. After a comparison with GMM, we found high measurement redundancy in LMM protocols, possibly resulting in relatively high allometry but low discriminatory performance, as opposed to correct discriminatory performance in GMM even after allometric correction. These findings suggest that taxonomic measurement protocols might benefit from GMM‐based pilot studies, because this offers the option of differentiating allometric and non‐allometric shape differences between species, which can then inform on the development of the easier‐to‐apply LMM protocols.

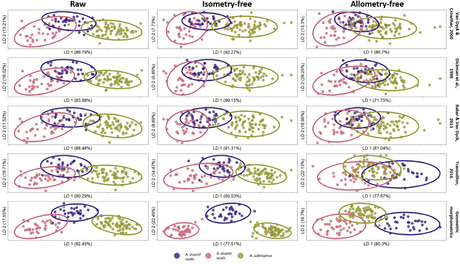

## 1 INTRODUCTION

Morphometric measurements are an important tool in efforts to differentiate mammalian species from each other and have been used in taxonomic research for centuries. Mammalian skulls in particular are widely used for taxonomic diagnostics and have long provided important data which can be used in the delimitation of species or Evolutionary Significant Units (ESUs). Cranial morphometric measurements are widely used in the separation of closely related mammalian groups around the world, ranging across disparate taxa such as rodents (Alhajeri, 2021; Boroni et al., 2017), bats (Schmieder et al., 2015), mustelids (Abramov et al., 2018; Gálvez‐López et al., 2022), whales (Rosel et al., 2017), and marsupials (Cáceres et al., 2016; Prevosti et al., 2012).

**FIGURE 1:**
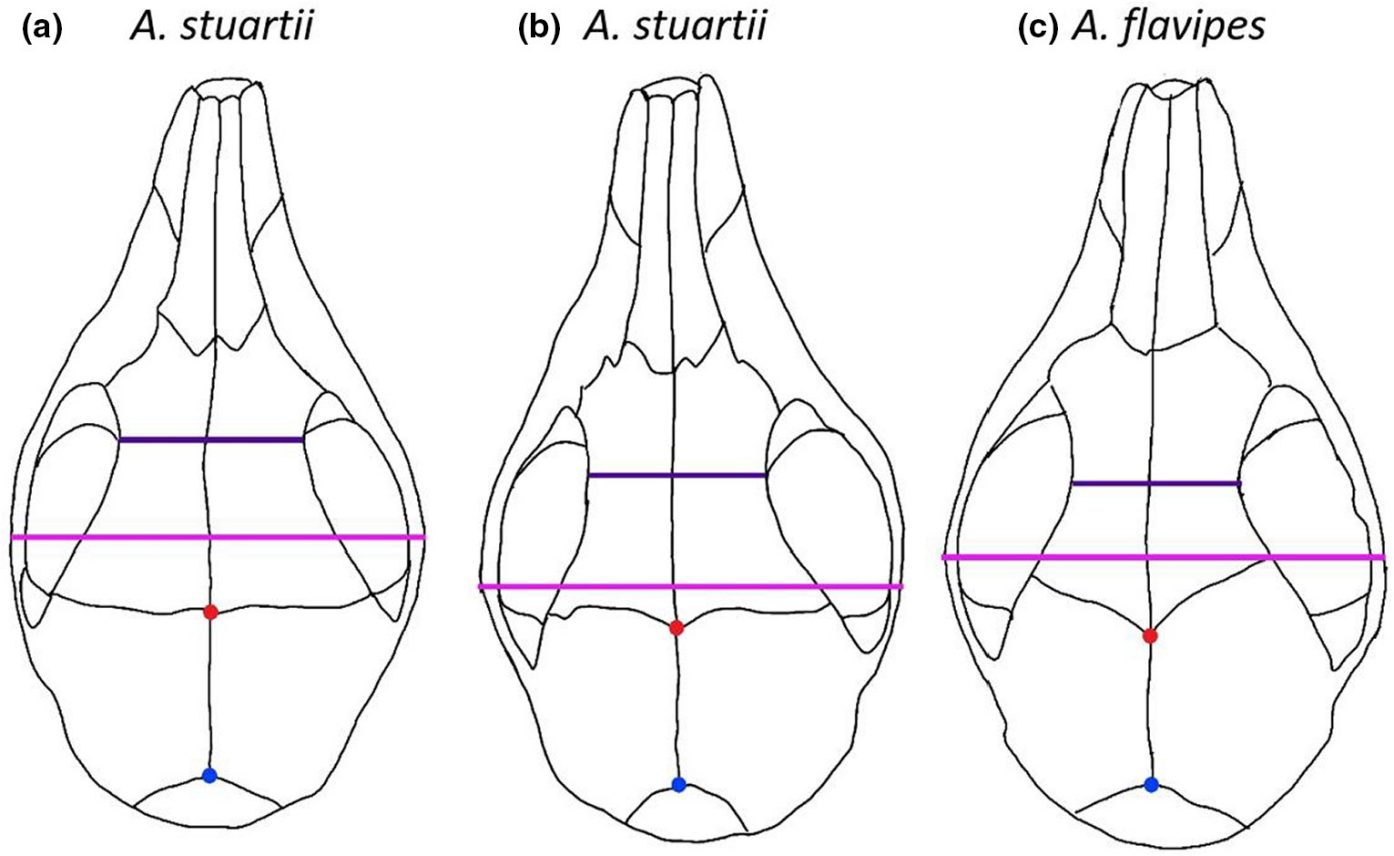
Three specimens (CM12785, CM6540 and CM10548) outlining two commonly used linear distances: the width of greatest constriction of orbitotemporal fossa (dark purple) and the maximum width of cranium measured across zygomatic arches (pink). In addition, two type I homologous landmarks (by suture intersection) are depicted: the fronto‐parietal suture in midline (red dot) and the parietal–interparietal suture in midline (blue dot). The two examples of maximum and minimum distances are measured at different anatomical positions relative to the homologous landmarks and other sutures in the skull, indicating a possibly serious lack of homology.

Morphometrics‐based taxonomic differentiation remains mostly the domain of linear morphometrics (LMM) (Jackson & Groves, 2015), where point‐to‐point distances are used to characterize and quantify differences between taxonomic units. Such linear measurements are easily taken and have a long history, but the information they contain has some important limitations. In particular, linear measurements only describe distances between two points, meaning that they contain limited information about overall shape. In addition, different measurement protocols are frequently used for different taxa, based on the morphological expertise of the taxonomist making the measurements. In a linear context, this makes it difficult to compare the shape variation between groups that have been acquired using different protocols. In addition, the linear distances measured in taxonomic diagnoses often include maximum and minimum dimensions of particular skeletal parts, such as minimum/maximum heights, widths, and lengths that are easily identifiable to the eye. However, when shapes differ, maximum or minimum distances may not be comparable among individuals, as the point‐to‐point distance may relate to different reference points that are not necessarily biologically homologous across taxa (Figure 1). Lastly, LMM protocols commonly consist of linear measurements that contain other linear measurements within them (e.g., multiple measurements along the longitudinal axis of the skull that all contain partial information about overall skull length). This results in redundant and dominant size information in the dataset and can lead to the impression that groups are differentiated in shape when they are really just differentiated by size. This is important because, ideally, the effect of size needs to be accounted for in taxonomic studies (Bartels et al., 2011; Lleonart et al., 2000; Sidlauskas et al., 2011). In particular, the frequent use of proportional ratios (e.g., skull length vs. width) is problematic because many vertebrate species display intraspecific allometry (Marcy et al., 2020), such that genetically similar individuals will differ in a ratio simply because they differ in size (Sidlauskas et al., 2011).

A potential refinement of LMM protocol development, which would address the issues outlined above, could be offered by the use of Geometric Morphometrics (GMM). GMM uses the coordinates of anatomical reference points as shape variables in a given dataset of specimens. Since the 1990s, this technique (Adams et al., 2004) has become the standard method for shape characterization in evolutionary and ecological morphometrics and has a very mature analytical toolkit (Fruciano, 2016; Fruciano et al., 2017). Unlike the point‐to‐point approach of LMM, GMM allows the holistic characterization of the biological specimens under study and a graphical output of shape variation (Klingenberg, 2016; Stone, 1997). GMM can address primary homology by using fixed homologous landmarks (e.g., suture intersections). In addition, it allows the assessment of curves and surfaces through semi‐landmarks (Gunz & Mitteroecker, 2013; Palci & Lee, 2019; Zelditch et al., 2012) without the abovementioned issue of relying on distance minima/maxima in LMM contexts. GMM also makes it comparatively easy to understand shape variation patterns with visualizations of the warped morphological variation alongside principal components or the examination of other drivers of variation (e.g., ecological) (De Mendoza & Gómez, 2022; Dunn & Avery, 2021; Meloro et al., 2017; Viacava et al., 2020, 2021). These visualizations can also provide visual comparisons of shape variation between species even if the landmarking protocols are not identical.

Geometric morphometrics is particularly useful in its explicit treatment of size. The Procrustes superimposition procedure inherent to GMM (Zelditch et al., 2012) allows the removal of the size component from the dataset by scaling all specimens to the same size, or isometric scaling. This procedure results in two components: a proxy for size called centroid size and a multivariate shape component (Kendall, 1989). These can then be used for analyses of allometry (shape changes disproportionate to size) in the form of a shape versus size regression (Klingenberg, 2016, 2022). This substantially improves on the issue of accounting for isometric and allometric variation, which can have serious implications for taxon delimitation (Sidlauskas et al., 2011). Allometric effects in particular can give an impression of species differentiation, when cranial allometry is generally present within most mammalian species (Cardini et al., 2015; Marcy et al., 2020; Viacava et al., 2020, 2021) and may not be related to morphological divergence due to a speciation event (Sidlauskas et al., 2011). Such allometric variation has been regarded as irrelevant to taxonomy. This is because, if shape differences were strictly due to size differences, they are likely to be the differences between small and large animals within a taxonomic group (Pilbeam & Gould, 1974; Seifert, 2008; Wood & Stack, 1980). In contrast, non‐allometric shape variation between taxonomic units are thought to be caused by independent adaptive processes such as are involved in species divergences (Gould, 1975; Huxley, 1932), and can even be related to phenotypic plasticity (e.g., Weisbecker et al., 2019). Thus, it is recommended to include allometric analyses in integrative taxonomic studies in order to interpret the shape variation and to properly delimit species (Cardini & Polly, 2013; Kaliontzopoulou et al., 2008; Outomuro & Johansson, 2017; Seifert, 2008; Sidlauskas et al., 2011; Viacava et al., 2020, 2021; Yazdi, 2014). However, even in a case where taxonomic differentiation is driven purely by selection for size and coincides only with allometry effects, this represents important information on the differentiation process and should be considered.

Geometric morphometrics has been successfully used in taxonomic studies, demonstrating its use in two‐ and three‐dimensional contexts and also highlighting the ability further explore the ecological sources of shape variation (Cáceres et al., 2016; Meloro et al., 2017; Moreira et al., 2020; Sansalone et al., 2015). However, the majority of taxonomic works are linear measurement‐based, probably because taking linear measurements is cheaper and easier to acquire and analyze, including the advantage of having a large body of literature on taxonomic measurements that has been widely used for centuries (Sidlauskas et al., 2011). In addition, GMM data acquisition can be more complex, requiring digitisation of either photographs or 3D specimen representations, which generally involve specialized equipment. The statistical analyses required are open‐access, very well developed and versatile, but also specialized and involve high‐dimensional data (Adams & Otárola‐Castillo, 2013; Klingenberg, 2011; Zelditch et al., 2012). Therefore, they may not be perceived to be as straightforward as the statistical toolkits used in LMM analyses. However, we can also fairly argue that linear measurements can easily be extracted from GMM data by measuring the distance between two landmarks, enabling a potential complementary approach.

Here, we argue that GMM analyses are a useful technique for executing or augmenting taxonomic studies because they tackle several limitations of LMM, as discussed above. We demonstrate this by comparing the performance on taxonomic differentiation of conventional, linear‐based morphometrics and 3D GMM in the *Antechinus stuartii*/*Antechinus subtropicus* species complex found in Eastern Australia between Southern Queensland and Northern New South Wales. This species complex includes three genetically differentiated taxonomic groups, which share subtle morphological differences as determined by a previous 3D GMM study (Viacava et al., 2021). The genus *Antechinus* contains a group of small insectivorous marsupials that have undergone several taxonomic discoveries and re‐descriptions in the past decade (Baker & Van Dyck, 2013a, 2013b, 2013c, 2015; Baker et al., 2012, 2014, 2015). The 3D GMM dataset of Viacava et al. (2021) thus represents an ideal scenario where finer‐grained differences (e.g., allometric effects or differences in non‐homologous measurements) need to be identified and interpreted with great care to understand their pertinence to group differentiation. In addition, three LMM protocols have been used in the genus *Antechinus* (Baker & Van Dyck, 2013b; Dickman et al., 1998; Van Dyck & Crowther, 2000), allowing an assessment of how important protocol choice can be to the delimitation of taxonomic units when compared with the more global GMM protocol. We add to this also a more generic protocol developed for bandicoots (Travouillon, 2016), with a particularly high number of linear measurements. We use linear discriminant analysis (LDA) to ask how well the four protocols and our GMM protocol perform, with particularly attention to the ability to capture size and allometry.

## 2 MATERIALS AND METHODS

All analyses are based on a 3D landmark coordinate dataset from Viacava et al. (2021), which includes high‐coverage 3D landmarked crania with 412 landmarks (82 fixed landmarks, 185 curved semilandmarks, and 145 surface semilandmarks) of 136 crania of adult individuals reconstructed from surface‐scanned virtual 3D images. These included specimens of *Antechinus subtropicus* (*N* = 68), *Antechinus stuartii* north (*N* = 30), and *Antechinus stuartii* south (*N* = 38). All the analyses were performed in R version 4.0.4 (R Core Team, 2021). The code and raw 3D data are available on Github (https://github.com/pietroviama/Viacavaetal_LMMvsGMM).

We identified four linear measurement protocols that represent morphometric methods commonly used in Australian mammal taxonomy but are also specific to *Antechinus*. These include a protocol used for a species contained in the species complex studied here, *A. subtropicus* (Van Dyck & Crowther, 2000), a sister species of the species complex studied here, *A. agilis* (Dickman et al., 1998), a species within the genus *Antechinus*, *A. flavipes* (Baker & Van Dyck, 2013b) and a comprehensive protocol that was developed for Peramelemorphians (bandicoots) (Travouillon, 2016). The last protocol is not necessarily expected to apply well to the genus *Antechinus* because it was designed for a different order of marsupials. However, we included it as a useful comparison of performance with the other three sets of linear measurements, representing one of the most comprehensive protocols in the morphometric study of Australian mammals. All of these protocols differ from each other but overlap in some measurements (Table 1).

**TABLE 1:** Degree of overlap of linear measurements between protocols. The linear morphometrics (LMM) protocols in the rows cover a fraction of the LMM protocols in the columns.

	Van Dyck and Crowther (2000) (%)	Dickman et al. (1998) (%)	Baker and Van Dyck (2013b) (%)	Travouillon (2016) (%)
Van Dyck and Crowther (2000)		61.9	66.67	27.27
Dickman et al. (1998)	100		72.22	40.91
Baker and Van Dyck (2013b)	92.31	61.9		31.82
Travouillon (2016)	46.15	42.86	33.33	

To obtain linear measurement data, we extracted the linear distances of each protocol that could be estimated most appropriately from the coordinates of the landmarks used for the GMM approach (Table S1). These measurements were not exactly the same as caliper measurements; however, we assume that slight inconsistencies between linear‐based and 3D landmark‐based distances are acceptable because they were taken in a consistent fashion and the representation of shape taken with the linear distances is not lost. We averaged right and left measurements whenever possible.

### 2.1 Isometry and allometry

In GMM, the isometric component of shape (i.e., the shape that changes in a 1:1 proportion with size) is removed from the dataset through the scaling procedure of the Procrustes superimposition. This step brings all specimens to the same size, producing “isometry‐free” shape coordinates and a centroid size (Dryden & Mardia, 2016; Klingenberg, 2016) for each specimen. Centroid size can be used subsequently as a proxy for specimen size. To approximate this effect in the LMM context, we used an approach that is analogous to centroid size extraction by deriving the geometric mean of all variables of a specimen as the equivalent of that specimen's centroid size and using log‐shape ratios (log_10_[measurement/geometric mean]) as isometry‐free shape variables. This ensures that both datasets can be analyzed in an approximately equivalent way with regards to size (Claude, 2013; Mosimann, 1970).

In order to assess the effect of allometry on shape variation, we regressed the Procrustes shape variation against the natural logarithm of centroid size using the “geomorph” (version 4.0.4) (Baken et al., 2021) function “procD.lm” (Adams & Collyer, 2016; Collyer et al., 2015). For LMM, we regressed the linear data against the natural logarithm of the geometric mean with the *lm.rrpp* function of the “RRPP” package (version 1.3.1) (Collyer & Adams, 2018). We considered both centroid size and geometric mean as proxies for size in the context of geometric and LMM. We also computed “allometry‐free” datasets for the classification analyses below, by using the residuals from the allometric regressions. In summary, three types of morphological data were obtained and analyzed for the LMM protocols and the GMM dataset: (1) raw 3D coordinates obtained from a partial Procrustes superimposition (in GMM, this involves translation and rotation without scaling) and raw LMM, (2) shape after Procrustes superimposition (GMM) and log‐shape ratios as explained above (LMM), and (3) allometry‐corrected shape for both. In the case of raw shape, this type of data is typically called “form” in GMM (shape plus size). However, for practical purposes, we will further call the types of morphological data explained above as “raw”, “isometry‐free” and “allometry‐free” shape, respectively. Allometric regressions were performed with 1000 permutations and *p*‐values were calculated using Goodall's *F*‐test (Goodall, 1991).

### 2.2 Ordination

To assess if the main variation of shape related to differentiation between species, we computed principal component analysis (PCA) for each treatment (raw, isometry‐free, and allometry‐free measurements) and each linear measurement protocol and GMM. PCA has a long tradition of being used in morphometric studies to assess between‐group differences and is useful to understand if these differences dominate the variation within the dataset. However, note that lack of differentiation between groups in PC1/PC2 space does not mean that the groups are not differentiated; PCA is agnostic to groupings, such that variation that differentiates a particular group can also be “smeared” across many principal components (Bookstein, 2015, 2017a, 2017b; Klingenberg et al., 1996; Strauss, 2010; Weisbecker et al., 2019).

### 2.3 Classification rule

To assess how well specimens are predicted to belong to each group for each dataset we considered, we kept 95% of the PC variance of each dataset as a cut‐off threshold, to subsequently perform a LDA. Four to twelve and 22–68 eigenvalues were needed to retain 95% of PC variance in our LMM and GMM datasets, respectively. We used the clade identity as a group factor and provided an equal prior on class membership to the three groups. We plotted the first two linear discriminant axes for each treatment (raw, isometry‐free, and allometry‐free measurements), and for each linear measurement protocol and the GMM protocol. Next, we used a machine learning model known as “leave‐one‐out cross validation” to calculate the posterior probability values of a specimen belonging to a group (Venables & Ripley, 2002). We used the “klaR” package for R (Weihs et al., 2005) to calculate several metrics of classification performance of the family of Garczarek's classification performance measures, using the “ucpm” function (Garczarek & Weihs, 2003). These include correctness rate (CR), accuracy (AC), ability to separate (AS), confidence (CF), and confidence for each class. The CR and AC values estimate the degree of validity (quality) of the LDA from the predicted values based on the true values. AS corresponds to the distance between the posterior values and the assigned groups and CF measures the degree of confidence to which the groups have been assigned—both AS and CF estimate the “certainty” of the result of the LDA (Dr. Karsten Luebke, personal communication). Finally, we predicted the identity of unidentified specimens (*n* = 32). For this, we predicted the PC scores of the unidentified specimens and then used the LDA model of our “isometry‐free” datasets assigning equal priors to predict the class provenance for each specimen (Table S1).

A simplified workflow of the data extraction and subsequent analyses of this study is provided in Figure S1.

## 3 RESULTS

### 3.1 Allometry

All LMM and GMM protocols included significant allometry (Table 2). However, the amount of shape variation attributable to allometry differed substantially between protocols, from 7.9% using Van Dyck and Crowther's (2000) linear measurement protocol, to over 25% using Travouillon's (2016) linear measurement protocol. Dickman et al. (1998), Baker and Van Dyck (2013b) and GMM identified a similar allometric effect of between 11% and 14% of shape variation explained by size (Table 2).

**TABLE 2:** Classification performance measures (Garczarek & Weihs, 2003) of the four linear measurement protocols and geometric morphometrics (GMM).

	Van Dyck and Crowther (2000)			Dickman et al. (1998)			Baker and Van Dyck (2013b)			Travouillon (2016)			GMM		
	Raw	Isometry‐free	Allometry‐free	Raw	Isometry‐free	Allometry‐free	Raw	Isometry‐free	Allometry‐free	Raw	Isometry‐free	Allometry‐free	Raw	Isometry‐free	Allometry‐free
Correctness Rate	0.904	0.875	0.838	0.904	0.853	0.765	0.875	0.86	0.757	0.882	0.853	0.662	0.926	0.86	0.581
Accuracy	0.759	0.686	0.55	0.779	0.681	0.516	0.741	0.687	0.5	0.764	0.712	0.328	0.843	0.761	0.286
Ability to Separate	0.855	0.763	0.654	0.87	0.784	0.707	0.837	0.793	0.665	0.879	0.858	0.597	0.943	0.983	0.879
Confidence	0.915	0.861	0.79	0.924	0.874	0.817	0.905	0.879	0.794	0.927	0.914	0.749	0.967	0.99	0.928
Confidence for each true class	North: 0.89	North: 0.773	North: 0.72	North: 0.889	North: 0.802	North: 0.794	North: 0.86	North: 0.776	North: 0.726	North: 0.892	North: 0.863	North: 0.806	North: 0.963	North: 0.961	North: 0.947
	South: 0.875	South: 0.807	South: 0.787	South: 0.886	South: 0.817	South: 0.835	South: 0.87	South: 0.819	South: 0.783	South: 0.926	South: 0.897	South: 0.754	South: 0.953	South: 0.997	South: 0.944
	Sub: 0.949	Sub: 0.931	Sub: 0.823	Sub: 0.961	Sub: 0.937	Sub: 0.818	Sub: 0.945	Sub: 0.958	Sub: 0.83	Sub: 0.944	Sub: 0.946	Sub: 0.721	Sub: 0.976	Sub: 0.998	Sub: 0.91
Allometry	*R* ^2^ = .079, *F* = 11.475, *p* = .001			*R* ^2^ = .144, *F* = 22.48, *p* = .001			*R* ^2^ = .113, *F* = 17.064, *p* = .001			*R* ^2^ = .251, *F* = 44.99, *p* = .001			*R* ^2^ = .132, *F* = 20.403, *p* = .001		

*Note*: For each protocol, the classification performance measures were computed with raw datasets, after size treatment, and after allometry correction. Allometric regression results are also indicated in the last row. North, *Antechinus stuartii* north; South, *Antechinus stuartii* south; Sub, *Antechinus subtropicus*.

It is worth noting that in Viacava et al. (2021), we tested for differences in allometric slopes between the groups and sexes and found no clear effect. This means that our tests on allometry in this study are not missing informative differences among the groups or the sexes related to differing allometric regression slopes.

### 3.2 Ordination

**FIGURE 2:**
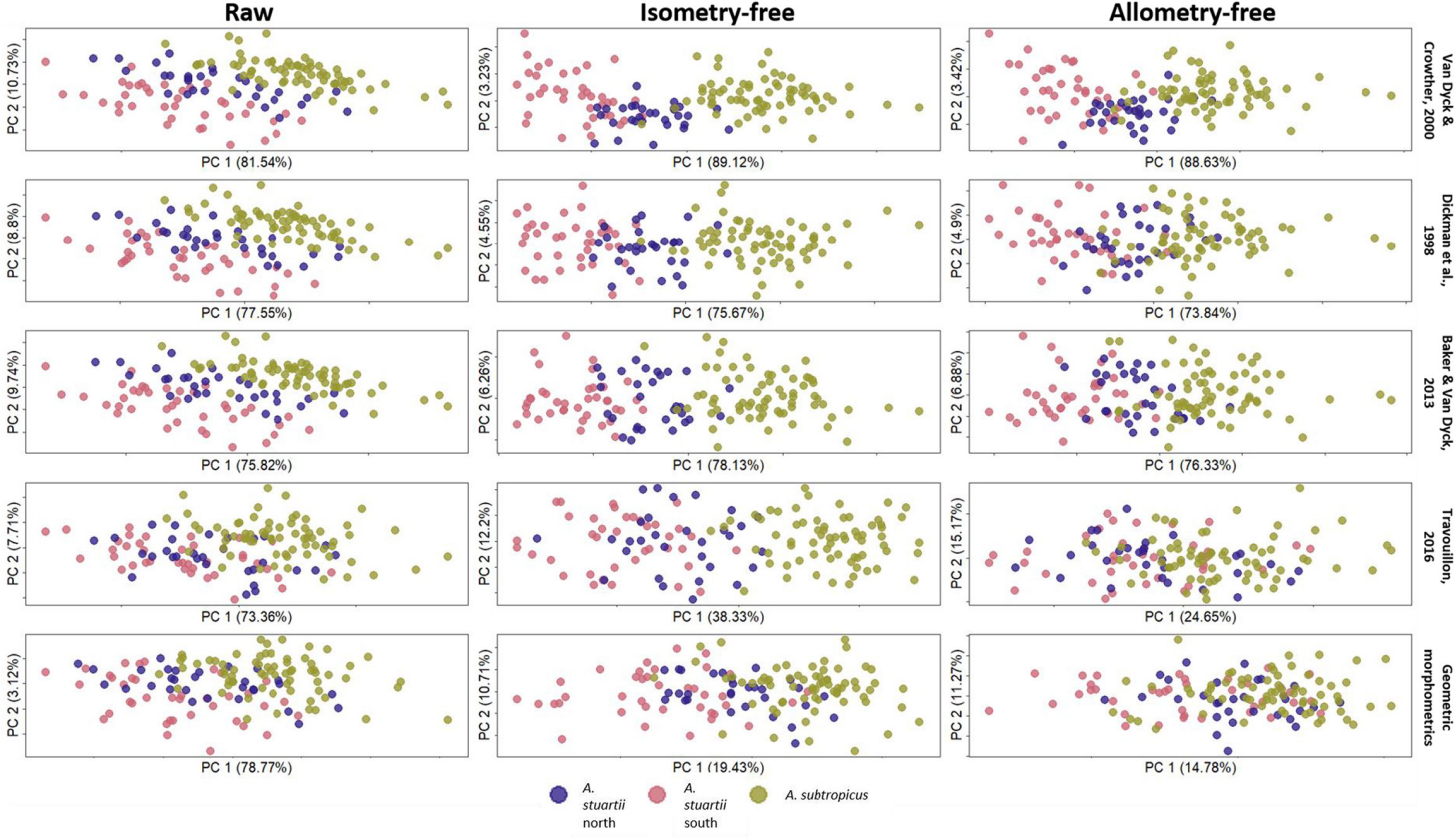
Principal component analyses plot for all raw, isometry‐free and allometry‐free datasets. These include the four linear measurement protocols and the geometric morphometrics approach. Only the first two principal components are shown.

The first principal component (PC1) of the three LMM protocols developed for antechinuses accounted for more than 70% of morphological variation in raw, isometry‐free and allometry‐free contexts (Figure 2). Travouillon's (2016) linear measurement protocol for bandicoot was a striking exception, dropping from 73.36% to 38.33% after correction for isometry and 24.65% after allometric correction. We also observed a reduction in morphological variation accounted by PC1 after removal of isometry in the GMM protocol, from 78.77% to 19.43%, and a slight decrease after allometric correction to 14.78%. Grouping of the three clades was affected in all three stages of data treatment; in all cases, isometry removal improved clustering of the groups along PC1, while allometric correction reduced the clustering effect (Figure 2).

### 3.3 Classification rule

**FIGURE 3:**
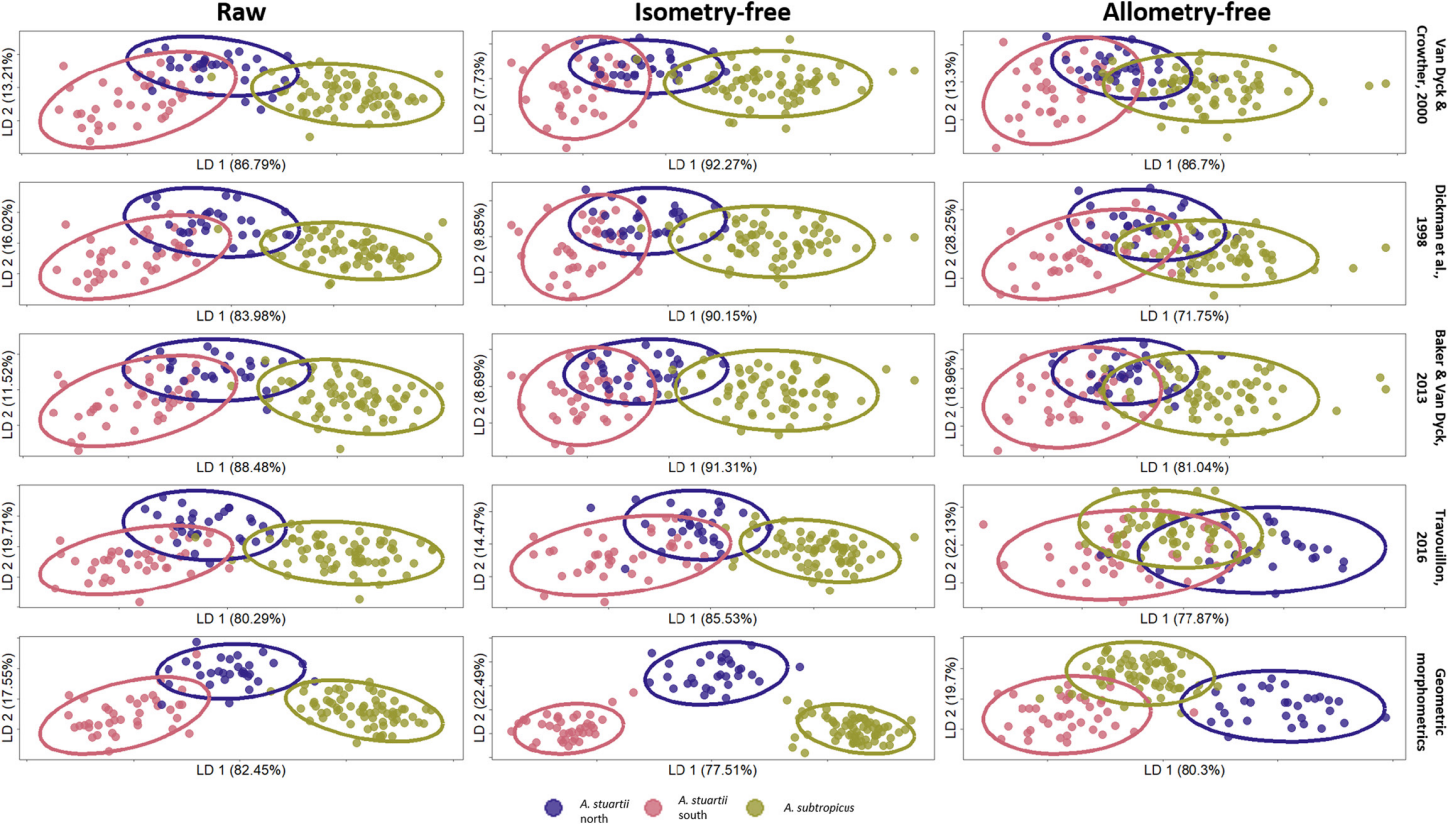
Linear discriminant analyses plot for all raw, isometry‐free and allometry‐free datasets used in this study. These include the four linear measurement protocols and the geometric morphometrics approach. Ellipses were computed at 95% confidence intervals.

The LDA plots display similar groupings of clades for raw and isometry‐free measurements in the LMM protocols. The removal of isometry increased group differentiation in the GMM protocol (Figure 3; Table 2). Interestingly, the removal of allometry showed a considerable decrease in group differentiation in the LMM protocols (Figure 3; Table 2). For the GMM protocol, the removal of allometry did not affect group differentiation as much as for LMM (Figure 3; Table 2).

For size‐unadjusted raw data, the classification performance measures were reasonably high in all four LMM protocols (Table 2). After isometry removal and allometric correction, these measures decreased to varying degrees for all LMM protocols. GMM performed better than LMM at group discrimination at the raw data stage. After the removal of isometry, GMM performed similarly to LMM protocols in CR and AC (“quality” measures) and better in AS and CF (“certainty” measures). After allometric correction, a large decrease in CR and AC was observed in GMM data despite similar performance in AS and CF.

## 4 DISCUSSION

Our results showed that LMM performed well in distinguishing the three closely related species of antechinus in our dataset. However, the confidence of differentiations was better for the GMM protocol, particularly after size correction. There is also a clear indication that measurement choice has a substantial influence on the discriminatory performance of a linear measurement protocol, highlighting the care with which measurements need to be chosen. In other words, some linear measurement protocols might seem ideal at discriminating, but this will only be the case if the linear distances selected are best at discriminating in “reality”. This is probably why the protocol developed for bandicoots (Travouillon, 2016), the only LMM protocol used in this study not optimized for antechinuses, had the lowest classification performance metrics among all protocols.

We found that GMM performed relatively better at discriminating groups based on raw and isometry‐free data, while the LMM protocols were highly dependent on the choice of the measurements. The fewer variables relative to GMM may therefore improve the discrimination of LMM protocols, but only if the selected linear distances are the “real” best discriminatory ones. In the case of 3D GMM, this dependence on measurement choice is expected to be less pronounced if the creation of the landmarking template relies on the agnostic and comprehensive placement of homologous reference points present in all specimens in a given dataset. The selection of the landmarks should therefore involve the construction of a template that attempts optimal anatomical coverage with diverse homologous points.

Visual display of the main variation (PC1 vs. PC2 plots; “PCA plots” from hereon in) highlights the important issue that an interpretation solely based on the first principal components can be misleading (Schreiber, 2021; Weisbecker et al., 2019) and in our case can lead to a misunderstanding on the performance of GMM data. For GMM data, the PCA plots revealed unclear grouping of the clades (see Figure 2), compared with the much clearer differentiation of clades for the LMM protocols. However, the classification performance measures that used 95% of PC variance of all protocols reflect the ability of GMM to differentiate among clades exceedingly well (see Table 2).

This superficially better group differentiation seen in the LMM PCA plots relative to the GMM PCA plot is chiefly due to the lower dimensionality of the LMM dataset and the fact that, in these particular cases, the linear distances were well‐chosen to reflect group differences as their main variation. However, this simply reflects the fact that the GMM dataset contains far more variation—and information on shape—overall, much of which does not differentiate clades. As PCA is agnostic to group membership, the principal components containing variation that discriminates groups did not dominate the dataset and were “hidden” in lower‐ranked PCs (Bookstein, 2017b; Klingenberg et al., 1996; Weisbecker et al., 2019). The relevance of ignored morphological variance in a PCA biplot in GMM is emphasized in our LDA results where 95% of the PC variance was considered. This showed a more similar or better performance of group discrimination in GMM compared with LMM protocols, relative to what we observed in the PCA plots.

The GMM protocol had an interesting property of numerically (and visually; Figure 3) increasing the “certainty” measures of classification after the isometry removal step (between raw and isometry‐free datasets). The contribution of GMM toward isometry‐free group separation may be a substantial improvement in the way we regard size and shape as independent variables for subsequent allometric analyses. In the case of GMM, the large number of landmarks may contribute to a holistic characterization of size in the form of centroid size (Mitteroecker & Gunz, 2009). In the LMM context, the linear distances capture the size of the skull less well (Farkas et al., 2002; Slice, 2006). For example, if we measured only the length or the width of a skull, other linear distances associated with size‐related shape could be ignored, such as the width of the snout. This can be a problem because it disregards measures that are characterizing the size of a three‐dimensional object (Adams et al., 2004). Furthermore, if size is not characterized well, further consequences on the independence of a size and shape variable can undermine allometric analyses in the form of a size versus shape regression (Klingenberg, 2016).

The removal of shape variation due to allometry (the step from “isometry‐free” to “allometry‐free”) mostly resulted in larger decreases in classification performance measures compared with the previous step of removal of isometry (from “raw” to “isometry‐free”). In the GMM dataset, this step of removal of allometry coincided with a greater decline in Correctness Rate and Accuracy but lesser decline in Ability to Separate and Confidence (see Table 2) compared with the LMM protocols. We draw two possible interpretations from this result. On one hand, the redundancy in the information of nearby landmarks and semilandmarks could result in “low quality” classification (low CR and AC). On the other hand, GMM could deal more effectively with allometric variation, resulting in a “more accurate” allometric correction and a “highly certain” classification (highest AS and CF among the datasets). We note that this result may be an indicator of the former where the LDA may wrongly assign classes with false “certainty” due to the poor ratio between variables (PC scores) and observations (number of individuals) typically encountered in GMM. However, we suspect that the latter is the case because the step of removal of allometry has a similar large decrease on Accuracy in GMM and Travouillon's protocol (2016), despite the much higher allometric effect captured by Travouillon's linear measurements. This “large amount of allometry” captured by Travouillon may be caused by the redundancy of some linear measurements that exacerbate some shape patterns driven by size, which results in a drastic reduction in Ability to Separate. However, this is not the case in GMM, where the Ability to Separate remains high after removal of allometry. These contrasting results suggest that GMM techniques provide a more thorough way of dealing with allometry‐driven shape patterns compared with linear measurements.

Our study suggests that GMM and its statistical toolkit provides improved insights into taxon discrimination and is particularly useful for nuanced assessment of allometric patterns. This is particularly important where groups have different cranial proportions but are in fact differently sized populations of an allometrically uniform group. Taxonomic “splitting” of such a group may of course still be warranted, but it can be done with a clear understanding that the split reflects differences in size, as opposed to fundamentally different proportions that exist outside of a common allometric pattern that still unites the groups in question. It is important to note that even non‐allometric variation is not always related to evolutionary differentiation because differences between species might arise purely from phenotypic plasticity, for example, when putative ESUs live in different habitats (Mitchell et al., 2018). For this reason, taxonomic study including genetic and ecological data linked to morphological data is important to support the hypothesis of an independent adaptive process from non‐allometric shape changes.

Geometric morphometric‐based taxonomic studies are an excellent avenue of providing nuanced information on diagnostic differences between groups of interest (Alhajeri, 2021; Boroni et al., 2017; Cáceres et al., 2016; Gálvez‐López et al., 2022; Prevosti et al., 2012). Our previous work on the dataset used in this study demonstrates this, as it allowed the identification of linear measurements that optimally differentiate the three groups of *Antechinus* investigated here (Viacava et al., 2021). Of course, GMM is not practical as a default for many investigators, because it is time consuming and requires specialist equipment, analytical, and acquisition expertise. However, our results show how strongly the efficiency of LMM protocols depends on the selection of appropriate measurements. GMM is therefore an excellent first “pilot” step to identify linear measurements that are most likely to discriminate best between potential groups. The investment of time and resources into such GMM pilot studies therefore seems worthwhile. These can ensure that morphometric data acquisition efficiently focus on measurements that account for the relevant shape patterns identified in GMM and be applied to larger sample sizes using simpler linear acquisition methods.

## AUTHOR CONTRIBUTIONS

**Simone P. Blomberg:** Formal analysis (equal); funding acquisition (equal); methodology (equal); supervision (equal); validation (equal); writing – review and editing (equal). **Vera Weisbecker:** Conceptualization (equal); funding acquisition (equal); resources (equal); supervision (equal); validation (equal); writing – review and editing (equal). **Pietro Viacava:** Conceptualization (lead); data curation (lead); formal analysis (lead); investigation (lead); methodology (lead); project administration (lead); resources (lead); software (lead); validation (lead); visualization (lead); writing – original draft (lead); writing – review and editing (lead).

## CONFLICT OF INTEREST STATEMENT

The authors declare no conflict of interest.

### OPEN RESEARCH BADGES

This article has earned Open Data, Open Materials and Preregistered Research Design badges. Data, materials and the preregistered design and analysis plan are available at [https://github.com/pietroviama/Viacavaetal_LMMvsGMM].

## Supporting information


Table S1.

Figure S1.

## Data Availability

All the data and coded analyses are available in Github (https://github.com/pietroviama/Viacavaetal_LMMvsGMM).
