# Supplementary material for: The relative performance of geometric morphometrics and linear‐based methods in the taxonomic resolution of a mammalian species complex
Source: Ecol Evol. 2023 Mar 28;13(3):e9698. doi: 10.1002/ece3.9698 (PMC10049884; doi:10.1002/ece3.9698)
Supplement: Supplementary file 1 — Table S1. [file ECE3-13-e9698-s002.docx]

Supp. Table 1: Class predictions for unidentified specimens using isometry-free data.

|  | Van Dyck & Crowther, 2000 | | Dickman et al., 1998 | | Baker & Van Dyck, 2013 | | Travouillon, 2016 | | Geometric morphometrics | |
| --- | --- | --- | --- | --- | --- | --- | --- | --- | --- | --- |
|  | Class | Posterior probability (%) | Class | Posterior probability (%) | Class | Posterior probability (%) | Class | Posterior probability (%) | Class | Posterior probability (%) |
| CM12785 | *A. subtropicus* | 99.87 | *A. subtropicus* | 99.9 | *A. subtropicus* | 99.66 | *A. subtropicus* | 99.81 | *A. stuartii south* | 99.64 |
| CM12786 | *A. stuartii north* | 84.95 | *A. stuartii north* | 81.17 | *A. stuartii north* | 88.92 | *A. stuartii north* | 90.51 | *A. stuartii north* | 100 |
| JM21536 | *A. stuartii north* | 85.38 | *A. stuartii north* | 92.07 | *A. stuartii north* | 93.57 | *A. stuartii south* | 68.17 | *A. stuartii south* | 95.16 |
| J3810 | *A. stuartii south* | 62.85 | *A. stuartii north* | 52.21 | *A. stuartii north* | 60.23 | *A. stuartii north* | 97.48 | *A. stuartii north* | 70.15 |
| CM3795 | *A. stuartii south* | 87.99 | *A. stuartii south* | 94.54 | *A. stuartii south* | 89.58 | *A. stuartii north* | 67.2 | *A. stuartii north* | 100 |
| J5030 | *A. stuartii south* | 93.87 | *A. stuartii south* | 93.94 | *A. stuartii south* | 91.17 | *A. stuartii south* | 86.6 | *A. stuartii south* | 100 |
| JM4432 | *A. stuartii north* | 82.92 | *A. stuartii north* | 92.35 | *A. stuartii north* | 92.28 | *A. stuartii north* | 97.41 | *A. stuartii north* | 100 |
| JM3944 | *A. stuartii north* | 92 | *A. stuartii north* | 98.69 | *A. stuartii north* | 93.86 | *A. stuartii south* | 90.03 | *A. stuartii north* | 100 |
| M22782 | *A. stuartii north* | 88.39 | *A. stuartii north* | 96.85 | *A. stuartii north* | 93.28 | *A. stuartii north* | 98.8 | *A. stuartii north* | 100 |
| M22784 | *A. stuartii north* | 93.36 | *A. stuartii north* | 90.94 | *A. stuartii north* | 95.2 | *A. stuartii north* | 99.72 | *A. stuartii north* | 99.99 |
| M22785 | *A. stuartii north* | 58.88 | *A. subtropicus* | 51.36 | *A. stuartii north* | 57.38 | *A. stuartii north* | 88.51 | *A. stuartii north* | 100 |
| J15888 | *A. stuartii north* | 51.45 | *A. stuartii south* | 51.48 | *A. stuartii north* | 59.4 | *A. stuartii north* | 91.51 | *A. stuartii north* | 100 |
| JM14417 | *A. stuartii north* | 63.98 | *A. stuartii north* | 81.44 | *A. stuartii north* | 63.89 | *A. subtropicus* | 61.98 | *A. stuartii north* | 100 |
| RT1 | *A. stuartii north* | 97.79 | *A. stuartii north* | 99.42 | *A. stuartii north* | 98.32 | *A. stuartii north* | 98.36 | *A. stuartii north* | 100 |
| JM1600 | *A. stuartii north* | 80.24 | *A. stuartii north* | 81.09 | *A. stuartii north* | 83.52 | *A. subtropicus* | 69.26 | *A. stuartii south* | 96.95 |
| JM1596 | *A. stuartii north* | 81.93 | *A. stuartii north* | 87.6 | *A. stuartii north* | 93.59 | *A. stuartii north* | 74.2 | *A. stuartii north* | 99.28 |
| J17400 | *A. subtropicus* | 100 | *A. subtropicus* | 100 | *A. subtropicus* | 100 | *A. subtropicus* | 100 | *A. subtropicus* | 100 |
| J17401 | *A. subtropicus* | 82.82 | *A. subtropicus* | 83.59 | *A. subtropicus* | 97.78 | *A. subtropicus* | 93.8 | *A. subtropicus* | 100 |
| J17402 | *A. subtropicus* | 100 | *A. subtropicus* | 100 | *A. subtropicus* | 100 | *A. subtropicus* | 99.97 | *A. subtropicus* | 99.49 |
| J17403 | *A. subtropicus* | 99.97 | *A. subtropicus* | 99.99 | *A. subtropicus* | 99.99 | *A. subtropicus* | 99.96 | *A. subtropicus* | 100 |
| J17406 | *A. subtropicus* | 99.38 | *A. subtropicus* | 99.72 | *A. subtropicus* | 99.9 | *A. subtropicus* | 86.44 | *A. subtropicus* | 100 |
| JM1420 | *A. stuartii north* | 83.45 | *A. stuartii north* | 93.65 | *A. stuartii north* | 78.72 | *A. stuartii north* | 94.95 | *A. stuartii north* | 100 |
| JM14415 | *A. stuartii north* | 79.26 | *A. stuartii north* | 86.65 | *A. stuartii north* | 88.53 | *A. stuartii north* | 64.21 | *A. subtropicus* | 99.84 |
| J20265 | *A. stuartii north* | 95.54 | *A. stuartii north* | 96.88 | *A. stuartii north* | 97.77 | *A. stuartii north* | 72.12 | *A. stuartii north* | 93.19 |
| MWA1 | *A. stuartii north* | 77.94 | *A. stuartii north* | 86.11 | *A. stuartii north* | 73.15 | *A. stuartii north* | 68.85 | *A. stuartii north* | 99.69 |
| MWA2 | *A. stuartii north* | 81.1 | *A. stuartii north* | 83.42 | *A. stuartii north* | 78.38 | *A. stuartii north* | 98.39 | *A. stuartii north* | 100 |
| CG1 | *A. stuartii north* | 88.79 | *A. stuartii north* | 76.56 | *A. stuartii north* | 88.12 | *A. stuartii north* | 75.14 | *A. stuartii north* | 100 |
| JM20761 | *A. stuartii north* | 69.88 | *A. stuartii north* | 72.83 | *A. stuartii north* | 85.39 | *A. stuartii north* | 66.84 | *A. stuartii north* | 100 |
| JM21357 | *A. stuartii north* | 83.37 | *A. stuartii north* | 85.79 | *A. stuartii north* | 88.05 | *A. stuartii north* | 96.27 | *A. stuartii north* | 100 |
| JM14452 | *A. subtropicus* | 62.93 | *A. stuartii north* | 58.51 | *A. stuartii north* | 70.93 | *A. subtropicus* | 95.9 | *A. stuartii north* | 99.73 |
| CM674 | *A. subtropicus* | 93.56 | *A. subtropicus* | 97.38 | *A. subtropicus* | 99.64 | *A. subtropicus* | 87.61 | *A. subtropicus* | 99.84 |
| CM675 | *A. stuartii north* | 96.48 | *A. stuartii north* | 97.83 | *A. stuartii north* | 97.61 | *A. stuartii north* | 93.34 | *A. subtropicus* | 100 |
