# Supplementary figures and images for: The relative performance of geometric morphometrics and linear‐based methods in the taxonomic resolution of a mammalian species complex
Source: Ecol Evol. 2023 Mar 28;13(3):e9698. doi: 10.1002/ece3.9698 (PMC10049884; doi:10.1002/ece3.9698)

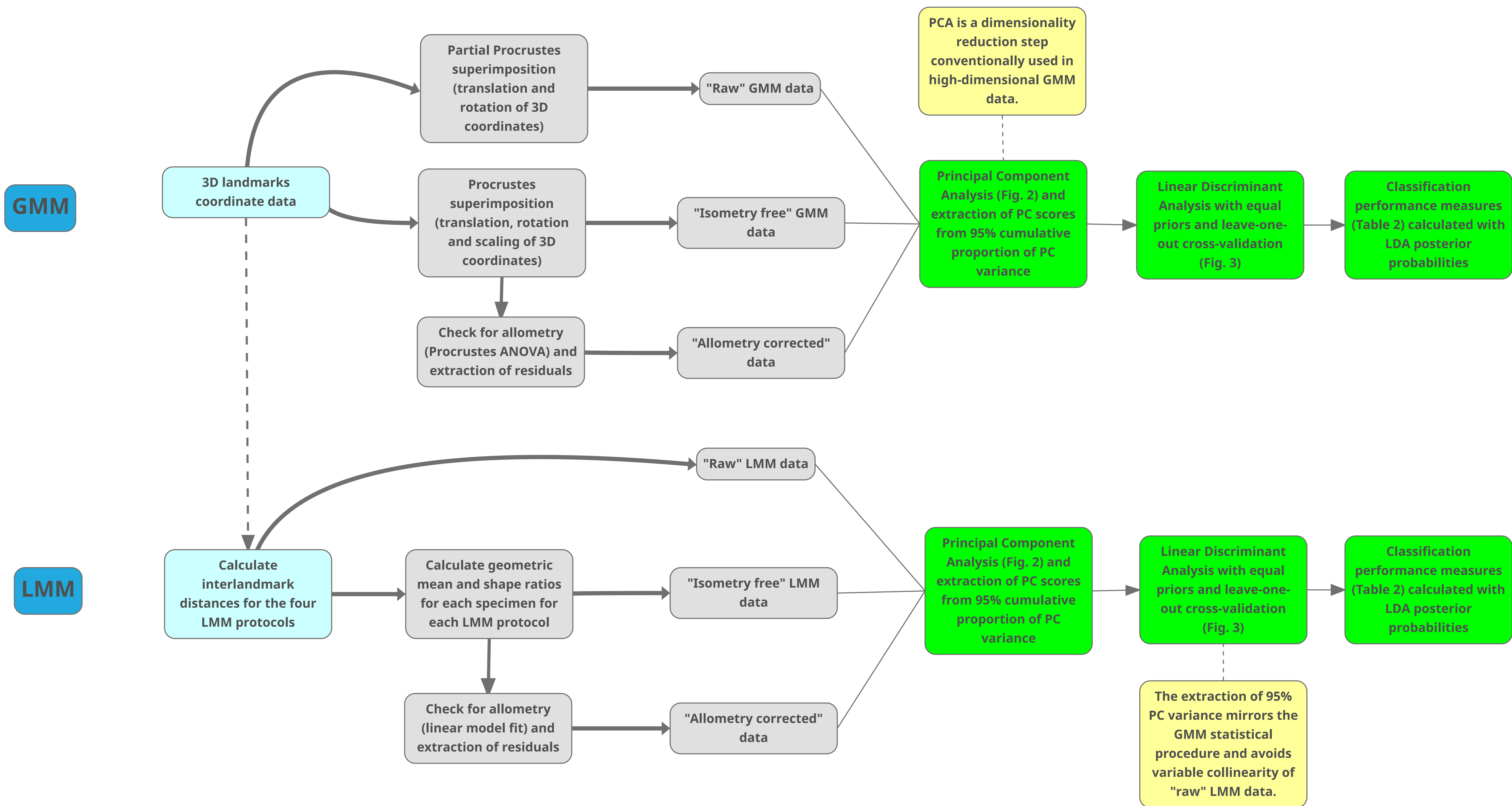

Supplement: Supplementary file 2 — Figure S1. [file ECE3-13-e9698-s001.pdf]
